# Impact of Oil Addition on Physicochemical Properties and In Vitro Digestibility of Extruded Pineapple Stem Starch

**DOI:** 10.3390/polym16020210

**Published:** 2024-01-11

**Authors:** Juthamath Nisitthichai, Phimraphat Wannaphruek, Jiratthitikan Sriprablom, Manop Suphantharika, Siwaporn Meejoo Smith, Taweechai Amornsakchai, Rungtiwa Wongsagonsup

**Affiliations:** 1 Division of Food Technology, Kanchanaburi Campus, Mahidol University, Kanchanaburi 71150, Thailand; juthamath.nis@alumni.mahidol.ac.th (J.N.); phimraphat.wan@alumni.mahidol.ac.th (P.W.); 2 Institute of Nutrition, Mahidol University, Nakhon Pathom 73170, Thailand; jiratthitikan.sri@mahidol.ac.th; 3 Department of Biotechnology, Faculty of Science, Mahidol University, Rama 6 Road, Bangkok 10400, Thailand; manop.sup@mahidol.ac.th; 4 Center of Sustainable Energy and Green Materials, Faculty of Science, Mahidol University, Nakhon Pathom 73170, Thailand; siwaporn.smi@mahidol.edu (S.M.S.); taweechai.amo@mahidol.ac.th (T.A.); 5 Department of Chemistry, Faculty of Science, Mahidol University, Nakhon Pathom 73170, Thailand

**Keywords:** pineapple stem starch, extrusion, oil addition, starch digestibility, physicochemical properties

## Abstract

The effects of palm oil (PO) and coconut oil (CO) additions on the physicochemical properties and in vitro starch digestibility of extruded pineapple stem starch (PSS) were studied. The native PSS was adjusted to 15% moisture and blended with PO or CO in amounts of 5 and 10% (*w*/*w* of starch), while the control sample without added oil was adjusted to 25% moisture before being extruded with a twin-screw extruder at a maximum barrel temperature of 140 °C. Due to the lubricating effect, the added oils reduced the expansion ratio of the extrudates, which led to an increase in cell wall thickness, bulk density, hardness, and water adsorption index, but to a reduction in the water solubility index, especially with 10% oils. PO had a greater impact on the physicochemical changes in the extrudates than CO. Surprisingly, no amylose-lipid complex was observed in the extrudates with added oil, as shown by XRD, DSC, and FTIR results. The phenolic compounds contained in PSS remained in all extrudates, which could affect the formation of the amylose-lipid complex during extrusion. The addition of 5% oil had no effect on the digestibility of the starch compared to the control extrudates, while the 10% oils, both PO and CO, reduced the rapidly digestible starch content but significantly increased the resistant starch content of the extruded PSS.

## 1. Introduction

The pineapple is one of the most important economic fruits grown in tropical and subtropical countries, including Thailand. The area under pineapple cultivation in Thailand is around 72,656 hectares, with an annual production of 1.68 million tonnes [1]. The fruit of the pineapple is eaten fresh or processed into various products, such as canned pineapple. The unprocessed parts of the pineapple that are left in the field, such as leaves and stems, become agricultural waste. In general, the disposal of this waste can be performed by sun-drying and incineration, which leads to environmental pollution [2]. Previous research has been carried out to increase the value of this agricultural waste in various ways. Pineapple leaf fibers have been used as thermal insulation, soundproofing, and plastic reinforcement [3]. Bromelain enzyme extracted from pineapple stems can be used as a meat tenderizer [4]. In addition, starch can also be extracted from pineapple stems with an extraction yield of 30% (dry basis) [2]. Pineapple stem starch (PSS) has unique and distinct properties compared to other commercially available native starches, such as a high amylose content (30–35%), high thermal stability, and a high content of slowly digestible starch and resistant starch [2,5]. It can be an alternative starch for the production of novel gluten-free extruded snacks from corn grit-PSS composite [6].

Extrusion is one of the food processing methods widely used by food manufacturers to produce various foods with unique texture and flavor characteristics [7]. It combines different operations, including mixing, kneading, shearing, cooking, and shaping [8]. The raw materials are processed under high mechanical shear force, high pressure, and high temperature in a short time to obtain the final product in the desired form [9]. Extrusion cooking leads to various chemical and structural changes, such as the gelatinization of starch, the denaturation of proteins, the formation of complexes between amylose and lipids, and the degradation of vitamins and pigments [10]. Most expanded extruded products are produced from starch-based materials, especially corn flour, as corn extrudates have a high expansion capacity and good sensory properties [6,11]. The expansion ratio increased with increasing amylose content of the corn flour in the feed mixture from 25% to 45% [12].

Lipids are common ingredients that act as lubricants and improve the flowability of raw materials during extrusion cooking [13,14]. They reduce the energy consumption required to extrude materials [15]. Extrusion cooking of common starch-based materials (such as corn starch, corn flour, and rice starch) with the addition of lipids (such as myristic acid, stearic acid, oleic acid, coconut oil, and fish oil) also changes the physical and chemical properties of the extrudates, such as reducing the expansion and water solubility index and increasing the product density, which is due to the formation of inclusion complexes between amylose and lipids [7,12,15,16]. The hydrocarbon portion of the lipids is located in the hydrophobic cavity of the single helical amylose and forms a V-type crystalline structure [17]. The amylose-lipid complex is resistant to enzymatic hydrolysis and has been proposed as a new source of resistant starch (type 5) [17]. Palm oil and coconut oil, as edible oils with a high content of saturated fatty acids, promote the formation of a stable amylose-lipid complex in the form of a single-helical inclusion complex, which limits the starch digestibility of cooked rice starch and rice flour [18]. These oils are also said to have various health benefits, such as reducing visceral adiposity and plasma glucose levels and lowering cholesterol levels [19,20,21]. The proportion of saturated fatty acids in palm oil and coconut oil is around 50% and 90%, respectively [22,23]. Saturated fatty acids are heat-stable and cannot be converted into trans fatty acids, which have a negative impact on health, causing coronary heart disease [24]. The extrusion process assisted in the formation of the amylose-lipid complex, with a higher yield obtained using saturated fatty acid [12,25].

In previous research, some studies have been conducted on the characterization [2,5,26] and modification of PSS [27], as well as on their applications in food [6] and non-food [28,29]. However, the study on the extrusion of PSS with oil additions has not yet been investigated. Therefore, the aim of this study was to investigate the effects of palm oil and coconut oil additions on the physicochemical properties and in vitro starch digestibility of extruded PSS. This research could be useful to expand the use of PSS from agricultural waste in food products in the future.

## 2. Materials and Methods

### 2.1. Materials

Pineapple stem residues, by-products from the bromelain extraction process, were obtained from Hong Mao Biochemicals Co., Ltd. (Rayong, Thailand). Palm oil and coconut oil were purchased from the Tops supermarket (Nakhon Pathom, Thailand). Palm oil consists of 49.9% saturated fatty acids, 39.2% monounsaturated fatty acids, and 10.5% polyunsaturated fatty acids. The fatty acid composition of palm oil includes 0.2% lauric acid, 1.1% myristic acid, 44.0% palmitic acid, 4.5% stearic acid, 39.2% oleic acid, 10.1% linoleic acid, 0.4% linolenic acid and 0.1% arachidic acid [22]. Coconut oil consists of about 90% saturated fatty acids and 10% unsaturated fatty acids. Coconut oil contains 0.50% caproic acid, 6.76% caprylic acid, 6.37% capric acid, 50.0% lauric acid, 17.19% myristic acid, 8.80% palmitic acid, 3.03% stearic acid, 5.25–10.54% oleic acid, 0.79–2.58% linoleic acid and 0.01–1.10% linolenic acid [23]. All chemicals used in this study were of analytical grade.

### 2.2. Pineapple Stem Starch Isolation

Pineapple stem starch was prepared according to the method of Nakthong et al. [2] with minor modifications. Pineapple stem by-products were mixed with distilled water in a ratio of 1:2 and blended with a mixer (Philips, model HR2118, Koninklijke Philips Electronics N.V., Amsterdam, The Netherlands). The slurry was filtered through cheesecloth to remove coarse fibrous material and then through 100-, 140-, and 270-mesh sieves to remove fine fibrous material. The filtrate fraction was centrifuged at 3000× *g* for 8 min. The supernatant was poured off, and the protein layer on the surface of the sediment was scraped off. The sediment fraction was washed several times with distilled water and centrifuged until the protein layer on the surface of the sediment disappeared. In the final step, the starch fraction was washed with 99.9% (*v*/*v*) ethanol solution and centrifuged again. The starch sediment was dried overnight in an oven at 45 °C. Finally, the starch was pulverized and sieved through a 100-mesh sieve and stored in a plastic bag.

### 2.3. Determination of Amylose Content

The amylose content of pineapple stem starch was determined using an amylose/amylopectin test kit (Megazyme International Ireland Ltd., Wicklow, Ireland). The method was briefly described by Sriprablom et al. [5].

### 2.4. Sample Preparation

The starch-lipid mixtures were prepared according to the methods of Cervantes-Ramírez et al. [25] and Tangsrianugul et al. [6] with some modifications. Pineapple stem starch was adjusted to 25% moisture for the control and 15% moisture for the oil-added samples by adding the appropriate amount of distilled water and blending for 5 min in a KitchenAid stand mixer (Hobart Manufacturing Co., Troy, OH, USA). Palm oil and coconut oil were then added in quantities of 5 and 10% (*w*/*w* of starch) and mixed for a further 5 min. The starch-lipid mixtures were then placed in sealed plastic bags and stored overnight at room temperature before being extruded. Pineapple stem starch without added oils was used as a control sample.

### 2.5. Extrusion Process

The starch-lipid mixture was extruded in a co-rotating twin-screw extruder (CTE-D22L32, Chareon Tut Co., Ltd., Samut Prakan, Thailand) with a die diameter of 4 mm. The diameter and length of the extruder screw are 21.5 and 700 mm, respectively. The extrusion conditions were chosen according to the preliminary data [6] and the equipment manufacturer’s guidelines to ensure good quality of the expanded snacks at normal recipes. The maximum barrel temperature used in this study was in accordance with the temperature used in the literature [30,31]. The barrel temperatures of six barrels were set to 40, 80, 120, 140, 120, and 120 °C, respectively. The die temperature was set to 140 °C. The screw speed was set to 450 rpm. The die pressure and torque were also monitored during the extrusion of the individual samples. After extrusion, the extrudates were dried in an oven at 80 °C for 15 min. The extrudates were cooled and kept in sealed plastic bags. Some of the extrudates were ground and sieved through a 60-mesh sieve. The ground extrudates were kept in sealed plastic bags and stored in a desiccator for further analysis.

### 2.6. Physical Properties of Extrudates

#### 2.6.1. Photograph

The photographs of the extrudates were taken with a digital camera (Nikon, model D7100, Nikon Corporation, Tokyo, Japan). The extrudates were cut with a razor blade with a height of 1 cm in cross-section. The cross-section and the whole piece of extrudate were placed on a black background before photographing.

#### 2.6.2. Expansion Ratio

The expansion ratio was determined according to the method of Shukri et al. [13]. The diameter of the extrudates was measured using a vernier caliper. The expansion ratio was determined for ten replicates using Equation (1).

Expansion ratio = d/d_die_ (1)

where d is the diameter of the extrudate and d_die_ is the diameter of the die.

#### 2.6.3. Bulk Density

The bulk density of extrudates was determined using the seed displacement method as described by Pardhi et al. [30] with a slight modification. The extrudates (10 g) were placed in a 250-mL measuring cylinder. The sesame seeds were placed in the measuring cylinder and lightly tapped ten times to replace the cavity. The volume of the sesame seeds was then measured and used to calculate the bulk density of the extrudates according to Equation (2) for three replicates.

(2) $$\mathrm{Bulk}\mathrm{density}\left(g/{cm}^{3}\right)=\frac{W}{250-V_{s}}$$

where W is the weight of the extrudates (g), and V_s_ is the volume of the sesame seeds (cm^3^).

#### 2.6.4. Hardness

The hardness of the extrudates was determined using a texture analyzer (TA.XT2i plus, Stable Micro Systems, Surrey, UK) with a blade set with a knife (HDP/BS). The test conditions were conducted according to the method of Tangsrianugul et al. [6]. The pre-test, test, and post-test speeds were set at 1, 2, and 10 mm/s, respectively, with a distance of 20 mm. The measurements were carried out on ten repetitions. The peak force at break was recorded as hardness.

#### 2.6.5. Water Absorption Index and Water Solubility Index

The water absorption index (WAI) and the water solubility index (WSI) were determined according to Shukri et al.’s method [13]. The ground extrudates (2.5 g) were suspended in 25 mL of distilled water in a 50-mL centrifuge tube. The mixture was then stirred continuously for 30 min at room temperature. The sample mixture was then centrifuged at 3000× *g* for 10 min. The supernatant was poured into an aluminum pan. The sediment in the tube was weighed. The supernatant was evaporated in an oven at 100 °C for 12 h, and the dry dissolved solids were weighed. Finally, the WAI and WSI were calculated using Equations (3) and (4), respectively.

WAI = W_sediment_/W_sample_ (3)

WSI = W_dry dissolved solids_/W_sample_ (4)

where W_sediment_ is the weight of the sediment, W_sample_ is the weight of the extrudates, W_dry dissolved solids_ is the weight of the dry solids of the supernatant.

#### 2.6.6. Thermal Properties

The thermal properties of the extrudates were analyzed using a differential scanning calorimeter (DSC) (model DSC-1 STARe System, Mettler-Toledo AG, Schwerzenbach, Switzerland). Ground extrudates (3 mg) were mixed with distilled water (9 mg) in a ratio of 1:3 in an aluminum pan and held at room temperature for 1 h before being placed in the DSC instrument. The sample pan was heated from 20 to 120 °C at a heating rate of 10 °C/min. The empty pan was used as a reference.

### 2.7. Structural Properties of Extrudates

#### 2.7.1. Scanning Electron Microscopy

The extrudates were cut with a razor blade in a radial cross-section with a thickness of 1 mm. The samples were mounted on aluminum stubs using double-sided adhesive tape and then coated with platinum under vacuum. The cellular structure of the extrudates was observed with a scanning electron microscope (SEM) (model JSM-IT200, JEOL Ltd., Tokyo, Japan) at an acceleration voltage of 20 kV.

#### 2.7.2. X-ray Diffraction

Native pineapple stem starch and ground extrudates were placed in a sample holder. The X-ray diffraction patterns of the samples were determined using an X-ray diffractometer (Empyrean Series 3, PANalytical B.V., Almelo, The Netherlands) at 40 kV and 40 mA and with Cu-K_α_ radiation of wavelength (λ = 1.5406 Å). The samples were scanned from 3 to 40° (2θ) using a step size of 0.0001°.

#### 2.7.3. Fourier Transform Infrared (FTIR) Spectroscopy

The FTIR spectra of the ground extrudates were analyzed using an FTIR spectrometer with attenuated total reflectance (ATR) (Frontier, Perkin Elmer, Waltham, MA, USA). The measurement was carried out from 4000 to 400 cm^−1^ with a resolution of 4 cm^−1^.

### 2.8. Chemical Properties

#### 2.8.1. Moisture Content and Water Activity (a_w_)

Each ground sample (2 g) was placed in an aluminum moisture can and analyzed for moisture content using a moisture analyzer (model MA100, Sartorius Thailand Co., Ltd., Bangkok, Thailand). The water activity (a_w_) of ground extrudates was determined using a water activity meter (Aqua-Lab model 4TE, METER Group, Inc., Pullman, WA, USA). The moisture content and water activity of the extrudates were determined in triplicate.

#### 2.8.2. Total Phenolic Content

The method for extracting the phenolic compounds was adopted from Kapcum et al. [32]. Native pineapple stem starch and ground extrudates (1 g) were extracted with 5 mL of 80% (*v*/*v*) ethanol in a 25-mL centrifuge tube and stirred continuously for 1 h at room temperature with a magnetic stirrer. The mixture was then sonicated with an ultrasonic cleaner for 30 min. The mixture was centrifuged at 4800× *g* for 20 min. The supernatant was filtered through Whatman filter paper No. 2 (GE Healthcare UK Limited, Buckinghamshire, UK).

The total phenolic content of the samples was determined according to the Folin–Ciocalteu method with a slight modification [32]. Briefly, the extract (125 µL) was mixed with 250 µL of Folin–Ciocalteu reagent and held for 5 min. To the mixture was added 25 mL of 7% (*v*/*v*) sodium carbonate and then allowed to stand for 90 min at room temperature. The absorbance of the mixtures was measured at 760 nm using a UV-VIS spectrophotometer (Lambda 25, PerkinElmer, Inc., Shelton, CT, USA). The TPC value was expressed as mg gallic acid equivalent per 100 g sample (mg GAE/100 g, db).

### 2.9. In Vitro Starch Digestibility

The in vitro starch digestibility of ground extrudates was determined according to the method described by Englyst et al. [33] with a slight modification. Ground extrudates (0.55 g) were mixed with 0.05 g guar gum and 20 mL 0.25 M sodium acetate buffer (pH 5.2). The enzyme solution containing porcine pancreatin and amyloglucosidase (5 mL) was added to the mixtures. The mixtures were then incubated in a shaking water bath at 37 °C with a stroke speed of 160 strokes/min. After 20 and 120 min, 0.25 mL of the mixtures were transferred to a centrifuge tube containing 10 mL of 66% (*v*/*v*) ethanol and mixed well to stop enzyme activity. The mixtures were centrifuged at 3000× *g* for 10 min. An aliquot of the supernatant (0.1 mL) was used to determine the released glucose using the GOPOD reagent assay kit (Megazyme International Ireland Ltd., Wicklow, Ireland). The starch fractions of rapidly digestible starch (RDS), slowly digestible starch (SDS), and resistant starch (RS) were calculated according to Equations (5)–(7), respectively.

%RDS = (G20 × 0.9 × 100)/TS(5)

%SDS = ((G120 − G20) × 0.9 × 100)/TS (6)

%RS = 100 − (%RDS + %SDS) (7)

where G20 and G120 represent the glucose released after 20 and 120 min, respectively, and TS is the total starch content.

### 2.10. Statistical Analysis

All measurements were carried out in triplicate, with the exception of the expansion ratio and hardness with ten replications. The results were reported as the mean ± standard deviation values. The significant difference (*p* ≤ 0.05) in the mean values was analyzed using analysis of variance (ANOVA) with Duncan’s new multiple range test. The correlation analysis between the various physical properties was determined using Pearson’s correlation test. The statistical analysis was carried out using SPSS version 21.0 (SPSS Inc., Chicago, IL, USA).

## 3. Results

### 3.1. Amylose Content of Pineapple Stem Starch

Pineapple stem starch (PSS) contains 31.24 ± 0.53% amylose content, which is consistent with Nakthong et al. [2] (35.15%) and Sriprablom et al. [5] (30.82%). The amylose content of PSS was significantly higher than that of commercially available starches, such as rice starch (21.46%), corn starch (22.88%), and tapioca starch (18.98%) [5]. Starch with a high amylose content (not exceeding 50%) has been reported to facilitate radial expansion of extrudates at a high extruder screw speed [12,34]. This high amylose content tends to be able to form a helical complex with lipids.

### 3.2. Physical Properties of Extrudates

#### 3.2.1. Visual Appearance

**Figure 1:**
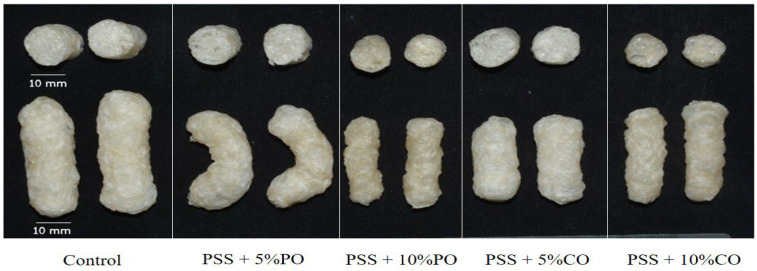
Photographs of extruded pineapple stem starch without (control) and with added oil at different levels; cross-section (top), whole piece (bottom) of the extrudates. PSS, pineapple stem starch; PO, palm oil; CO, coconut oil.

The visual appearance of the extrudates in the whole piece and in cross-section is shown in Figure 1. The control extrudates had the largest cross-sectional expansion. As the oil content increased, the extrudates became denser, and the diameter of the cross-sectional expansion of the extrudates decreased. This is consistent with the results of the expansion ratio, as described below. The control extrudates showed a yellowish to brownish color, which could be due to the presence of phenolic compounds in the PSS, as shown by the result of the total phenolic content (see Section 3.4.2). The extrudates become darker as the amount of added oils increases, which could be due to the extent of the browning reaction and the less-expanded structure. The phenolic compounds are oxidized at high temperatures, which is responsible for the browning reaction [35]. Color changes could be due to the occurrence of browning reactions, the degree of cooking, and the destruction of heat-sensitive pigments that take place during extrusion cooking [36].

#### 3.2.2. Expansion Ratio

The expansion ratio of extrudates without and with added oil in different quantities is shown in Table 1. The expansion ratio of the control extrudates was 4.41, which was the highest value. The expansion ratio of the extrudates decreased significantly with the addition of oils. With an oil addition of 10%, the extrudates had a lower expansion ratio than those with 5% oil. Depending on the type of oil added, the palm oil had a greater influence on reducing the expansion ratio of the extrudates than the coconut oil with the same amount of oil added. The radial expansion depended on the degree of cooking of the starch material [10]. The addition of lipids could normally retard the gelatinization of starch and change the dough rheology and consequently reduce the expansion of extrudates [10,12]. Palm oil contains a higher proportion of unsaturated fatty acids than coconut oil [22,23], which could lead to a better lubricating function, better lubricity of the raw materials, and a lower crushing effect during extrusion cooking and, consequently, a lower degree of starch gelatinization.

#### 3.2.3. Bulk Density

The bulk density of all extrudates is presented in Table 1. The control extrudates had the lowest bulk density (0.11 g/cm^3^). Both lipids increased the bulk density of the extruded PSS. The bulk density increased with increasing lipid content. Among the oils, palm oil had a greater influence on increasing the bulk density of extrudates than coconut oil. The bulk density was inversely correlated with the expansion ratio (*r* = −0.856, *p* ≤ 0.01) (Table 2).

#### 3.2.4. Hardness

The hardness of PSS extrudates with and without oils is summarized in Table 1. The addition of oils increased the hardness of the extrudates, especially when 10% oil was added. Here, too, palm oil had a greater effect on increasing the hardness of the extrudates than coconut oil. The hardness was positively correlated with the bulk density (*r* = 0.922, *p* ≤ 0.01) and inversely correlated with the expansion ratio (*r* = −0.880, *p* ≤ 0.01) of the extrudates (Table 2). When the expansion ratio decreases, bulk density and hardness increase as a result of the addition of oil. Due to the lubricating function of oil, it can reduce the grinding efficiency of raw materials, as shown by the reduction in torque with increasing oil content (Table 3), and consequently produce the hard texture of extrudates [14].

#### 3.2.5. Water Absorption Index and Water Solubility Index

The water absorption index (WAI) and the water solubility index (WSI) of extrudates with and without added oil in different levels are presented in Table 1. The WAI depends on the presence of intact molecules that do not lose their ability to bind water after extrusion [36]. The result showed that the WAI of extrudates with added oil was higher than that of the control extrudates. Moreover, the WAI increased with increasing oil addition. On the contrary, the WSI was inversely related to the WAI (*r* = −0.980, *p* ≤ 0.01) (Table 2). The extrudates with oils had a lower WSI than the control extrudates, especially the extrudates with 10% oils. The WSI is used as an indicator for the degradation of the molecular components during extrusion [36]. In addition to starch gelatinization, starch degradation, and dextrinization can lead to the formation of low molecular weight compounds that affect the increase in WSI [37]. As discussed above, lipids, in general, could retard the gelatinization of starch so that more intact starch molecules and fewer degraded molecules could be present in the extrudates with added oil, resulting in a higher WAI and a lower WSI of the extrudates with added oil compared to the extrudates without oil. At higher feed moisture (25%) and without lipid addition, the control sample could show a greater degree of starch gelatinization and degradation during extrusion cooking. The smaller molecules of the degraded starch could be more soluble in water. However, the type of oils had no significant effect on the WAI and WSI.

### 3.3. Structural Properties of Extrudates

#### 3.3.1. Scanning Electron Microscopy

**Figure 2:**
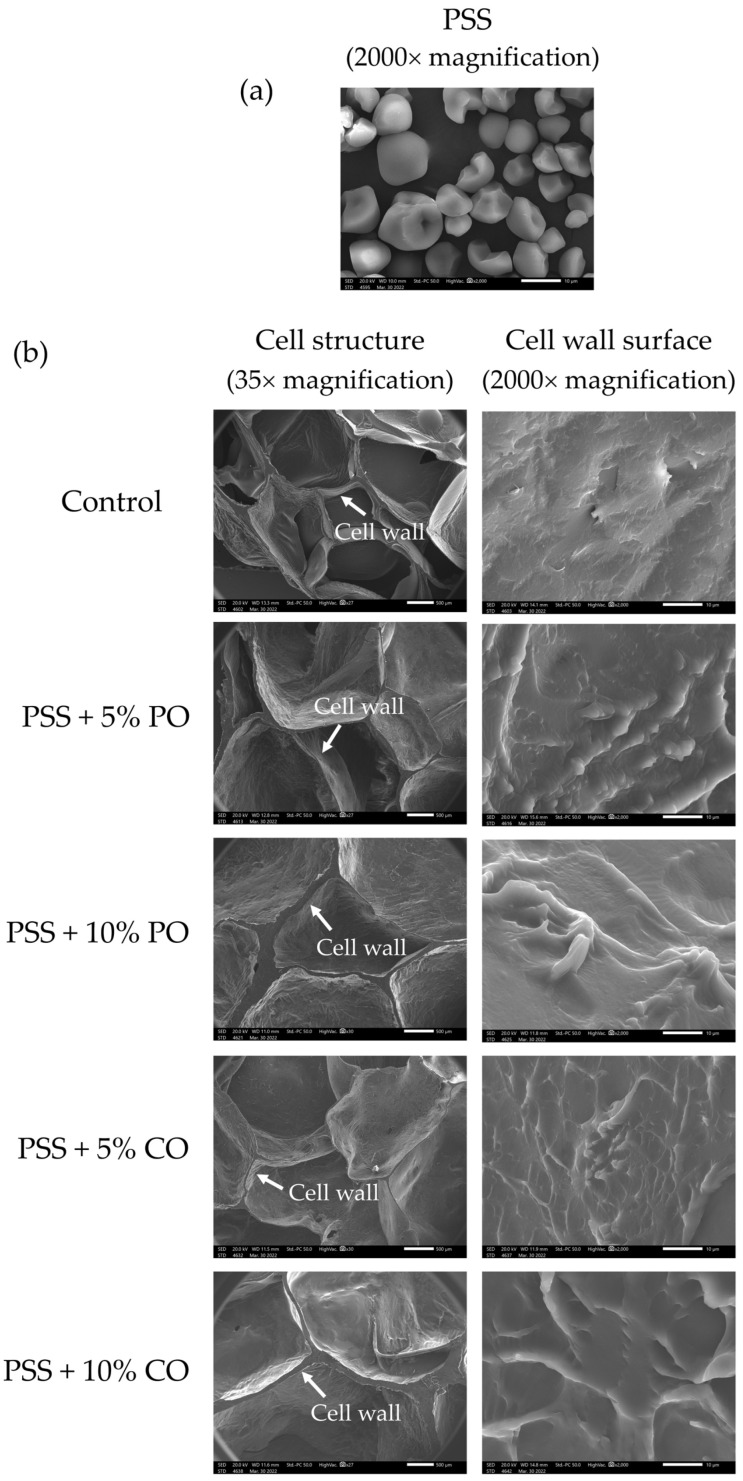
SEM micrographs of (**a**) native pineapple stem starch and (**b**) extruded pineapple stem starch without (control) and with added oil at different levels. PSS, native pineapple stem starch; PO, palm oil; CO, coconut oil.

Figure 2a shows a scanning electron microscope (SEM) image of native PSS (2000×). Native PSS granules had an asymmetrical shape. One side had a round shape and a smooth surface, while the other side had a polyhedral shape with several planes. Similar granular shapes of PSS were observed by Nakthong et al. [2] and Tangsrianugul et al. [6], who reported that the PSS granules have a semicircular shape with partially rounded segments and two or more plane surfaces.

Figure 2b presents the SEM images of the cell structure and cell wall surface of extruded PSS without (control) and with different levels of added oils. The cell structure of the control extrudates at a magnification of 35× showed a larger number of air cells and a thinner cell wall compared to extruded PSS with oils. Between the levels of oil added, the addition of 10% oil resulted in a larger cell wall thickness of the extrudates than the addition of 5% oil. In addition, the number of air cells decreased, but the size of the air cells increased in extrudates with 10% oil added compared to the extrudates with 5% oil and the control extrudates. The oils could interfere with the formation of air bubbles during extrusion by causing an uneven distribution of water in the material. The presence of substances such as lipids can change the elastic character of the melted starch so that it can no longer hold water vapor, resulting in cell wall rupture and reduced expansion [12]. The limited expansion is related to the large cell wall thickness of extrudates with oils. Su and Kong [14] reported that oil could function as a lubricant in extrusion cooking and reduce the grinding effect in the raw materials, which could lead to the formation of a thicker cell wall and a lower cell number of extrudates with oils. The lower initial moisture content (15%) of the oil-added samples could also limit water evaporation and the formation of air bubbles. Regarding the surface of the cell wall at a magnification of 2000×, the control extrudates showed a smooth cell wall surface without granular starch structure, indicating that the starch granules were completely gelatinized due to the high temperature, pressure, and mechanical shear during extrusion cooking [6]. Meanwhile, extrudates with palm oil and coconut oil showed a rougher and wavy cell wall surface, which could be due to the oil coverage on the surface. There was also no granular starch structure on the cell wall surface of extrudates with oils, indicating complete gelatinization of PSS.

#### 3.3.2. X-ray Diffraction

**Figure 3:**
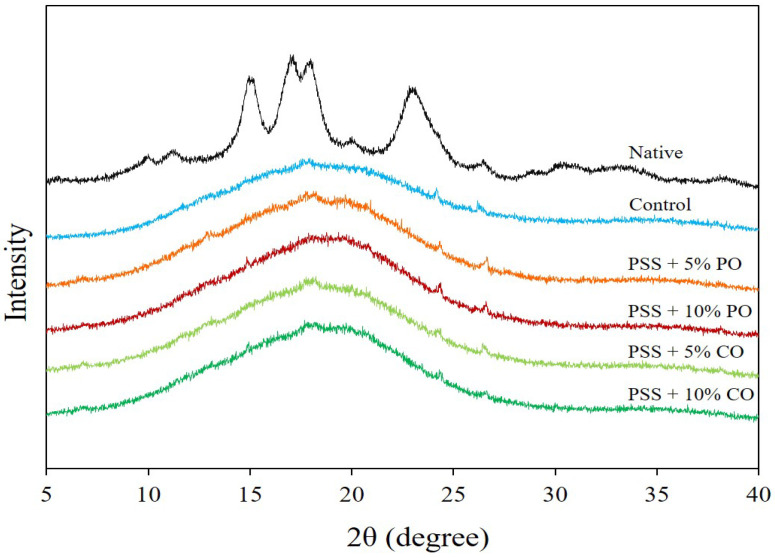
X-ray diffraction of native pineapple stem starch and extruded pineapple stem starch without (control) and with added oil at different levels. PSS, pineapple stem starch; PO, palm oil; CO, coconut oil.

Figure 3 shows the X-ray diffraction (XRD) pattern of native PSS and extruded PSS without (control) and with added oil at different levels. The native PSS showed the XRD peaks at positions 15.0, 17.0, 18.0, and 23.0° (2θ), which corresponded to the A-type crystal structure of starch, consistent with the previous report by Tangsriaugul et al. [6]. After extrusion, the A-type diffraction peaks of PSS disappeared, indicating complete gelatinization of the starch. The XRD pattern showed an amorphous background for all extrudates, mostly without a distinct peak. Surprisingly, the V-type pattern with peaks at 7.5, 13.0, and 20.0° (2θ) [25,38] was not found in extrudates with added oil. This indicates that PSS with oils was unable to form an amylose-lipid complex in the form of a single-helical inclusion complex under extrusion conditions. This could be due to the presence of phenolic compounds that remain in the PSS after starch extraction (see Table 3) and could interact with either starch or lipids [39,40], limiting the formation of the amylose-lipid complex. The phenolic compounds may interact with amylose via non-covalent interactions [39]. The pineapple stem contains a considerable amount of polyphenolic compounds (lignin) [41], which, due to their large molecular size, may not be able to penetrate the hydrophobic cavity of the single helical amylose and, therefore, form a non-inclusion complex with the amylose, preventing the formation of the amylose-lipid complex. Another reason is that the bulky molecular size of the oils causes steric hindrances and low water solubility, which prevents the formation of an inclusion complex between starch and triglycerides [25,42,43]. The formation of a starch-oil complex might require more severe processing conditions to achieve structural change of starch and triglycerides [25]. Noticeably, the tiny peaks at 24.3 and 26.5° (2θ) were observed in the control extrudates and the extrudates with oils, which might be related to the inherent phenolic compounds in PSS. Huang et al. [44] also found diffraction peaks at 24.3 and 26.1° (2θ) for cooked potato starch incorporated with dandelion flavonoids. The presence of free lipids was not detected in the XRD patterns [38].

#### 3.3.3. Thermal Properties

**Figure 4:**
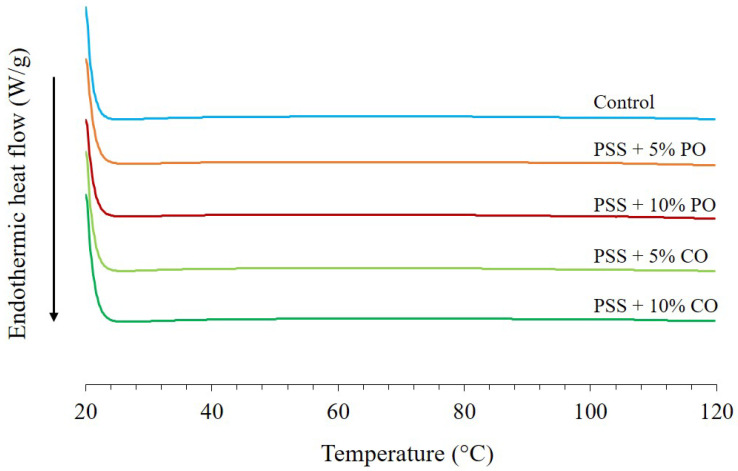
DSC thermograms of extruded pineapple stem starch without (control) and with added oil at different levels. PSS, pineapple stem starch; PO, palm oil; CO, coconut oil.

Figure 4 shows the DSC thermogram of extruded PSS without (control) and with added oil at different levels. There was no peak of starch gelatinization (below 100 °C), indicating that the extrusion process caused complete starch gelatinization, which is consistent with the SEM image shown in Figure 2b. In addition, the DSC thermogram of extrudates with added oils, both palm oil and coconut oil, showed no dissociation peaks of the amylose-lipid complex at 80–100 and 100–120 °C (dissociation temperatures of forms I and II of the amylose-lipid complexes, respectively) [38], which was consistent with the XRD pattern and confirmed that there was no V-type structure of the amylose-lipid inclusion complex. Phenolic compounds could interfere with the formation of the amylose-lipid complex so that the lipids cannot be inserted into the single helix of the amylose. Polyphenols could also interact with lipids, and this interaction could reduce fat absorption in the gastrointestinal tract, which could have a positive effect on health [40].

#### 3.3.4. FTIR Spectra

**Figure 5:**
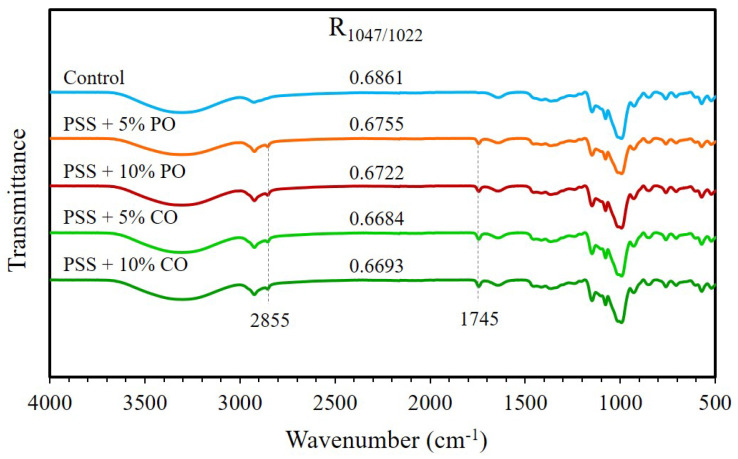
ATR–FTIR spectra of extruded pineapple stem starch without (control) and with added oil at different levels. PSS, pineapple stem starch; PO, palm oil; CO, coconut oil.

The FTIR spectra of all extrudates are shown in Figure 5. All extrudates exhibited absorption peaks at 3307, 2925, 1639, and 1149 cm^−1^, which were assigned to O–H stretching, C–H stretching, C-O bending associated with the OH group, and asymmetric stretching of the C–O–C group, respectively [45,46,47]. These peaks indicate the characteristic bands of starch. The peaks at 2855 and 1745 cm^−1^ were obviously observed in extrudates with added oils. These vibrational peaks, known as specific absorption bands of the fatty acids, correspond to the vibrations of the -CH_2_ and carbonyl groups of the fatty acids, respectively [25,48]. It showed that none of the oil molecules had entered into the helical hydrophobic cavities of the amylose and exhibited the IR absorption of these oils extensively. Guo et al. [48] reported that there are no obvious absorption bands of fatty acids in the FTIR spectra of starch-fatty acid complexes, suggesting that the fatty acid molecules have been inserted into the amylose single helix. The absorption values at 1047 and 1022 cm^−1^ are associated with crystalline and amorphous structures of starch [46]. The band at 1047 cm^−1^ was associated with the ordered structure of the inclusion complex [49]. The absorption ratio of 1047/1022 cm^−1^ (R_1047/1022_) can be used to study the short-range ordered structure of starch [47]. The R_1047/1022_ value of all extrudates with added oils was slightly lower than that of the control extrudates. This meant that oils could not improve the short-range order of starch via the formation of amylose-lipid complexes, which was consistent with the results of XRD patterns and DSC thermograms.

### 3.4. Chemical Properties of Extrudate

#### 3.4.1. Moisture Content and Water Activity

The moisture content and water activity of all extrudates are shown in Table 1. The moisture content of all extrudates was in the range of 6.47–7.62%, which is not a significant difference. This indicates that extrudates are food with a low moisture content. Even though the initial moisture content of the raw materials of the control sample (25%) was higher than that of the samples with added oil (15%), the moisture in the control sample evaporated faster than in the samples with added oil, resulting in similar final moisture contents of all extrudates with and without oils, and a higher expansion of the control extrudate was obtained. The water activity of all extrudates was in the range of 0.45–0.48, which is less than 0.6. Similar results were reported by Tangsrianugul et al. [6]. Therefore, the microorganisms cannot grow with limited water activity. All extrudates could be safe from microbial deterioration.

#### 3.4.2. Total Phenolic Content

The total phenolic content (TPC) of the native PSS and all extruded samples is shown in Table 3. The TPC in the native PSS was 53.93 mg GAE/100 g (db). This is probably due to the presence of a considerable amount of lignin, a polyphenolic compound, in the pineapple stem [41]. After extrusion, the phenolic compounds remained in the extrudates but in lower amounts than in the native PSS, which could be due to the destruction of the heat-sensitive phenolic compounds. The TPC of the control extrudate was significantly higher (*p* ≤ 0.05) than that of the extrudates with added oil, which was related to the extruder torque and die pressure. The higher initial moisture content of the control sample reduced the viscosity and shear of the plasticized mass in the extruder, resulting in lower extruder torque and die pressure and, consequently, lower degradation of TPC compared to the extrudates with added oil. When comparing the levels of oil added, extrudates with 10% oil showed a higher TPC value than extrudates with 5% oil. A high amount of oils caused a decrease in extruder torque and die pressure, as shown in Table 3. Oil acts as a lubricant that can reduce shear stress and heat build-up in the extruder, resulting in a reduction in material temperature and extruder torque [50]. Phenolic compounds could be less degraded at lower material temperatures with a 10% oil addition. The reduction in die pressure can be directly linked to the lower viscosity of the material due to the lubrication effect [50]. Palm oil has a higher proportion of unsaturated fatty acids, which can have a greater lubricating effect. The remaining phenolic compounds can interfere with the formation of the amylose-lipid complex, as the phenolic compounds can interact with the amylose instead via non-covalent interactions, such as hydrogen bonds and hydrophobic interactions [39,51]. Phenolic compounds can interact with starch via both V-type inclusion complexes and non-inclusion complexes, depending on the molecular size and structure of the phenolic compounds [52,53]. Small polyphenol molecules, such as anthocyanins and catechins, were able to form the V-type inclusion complex with starch [47,54], while large polymeric molecules of phenolic compounds, such as tannins, formed non-inclusion complexes via extensive hydrogen bonding and hydrophobic interactions [55]. In our case, lignin could interact with starch via a non-inclusion complex, as the lignin molecules are possibly too bulky and, therefore, cannot penetrate the hydrophobic cavity of the starch helix.

### 3.5. In Vitro Starch Digestibility

Table 4 shows the in vitro starch digestibility of all extrudates. Even without added oil, the control extrudates had a rather high RS content (12.93%), which could be due to the high amylose content of PSS [5]. The RDS, SDS, and RS contents of extrudates with 5% added oil did not differ significantly from those of the control extrudates. However, the extrudates with 10% added oil had significantly lower RDS but higher RS contents (*p* ≤ 0.05), while their SDS content did not differ significantly (*p* > 0.05) from the control and 5% added oil extrudates. These results of starch digestibility corresponded to the amount of phenolic compounds given in Table 3. The remaining phenolic compounds in extrudates with 10% oil addition were higher than in extrudates with 5% oil added. Phenolic compounds, such as persimmon tannins and dandelion flavonoids, may interact with starch via non-covalent interactions, leading to a reduction in starch digestibility [44,56]. In addition, the more compact structure with the thickest cell wall of the extrudates with 10% oil added also had the effect of limiting the susceptibility of the starch to enzyme hydrolysis. The oil can also hinder the access of the amylolytic enzymes to hydrolyze the starch molecules by covering the cell wall surface of the extrudates with added oils, as observed in the SEM (Figure 2). Therefore, the highest RS content and the lowest RDS content were found at 10% oil added. RS is likely to be inversely correlated with the glycemic index. Foods with a higher RS are digested slowly, resulting in a slower release of glucose and, consequently, a lower glycemic index and insulin response compared to foods with a low RS [57].

## 4. Conclusions

Different types and additional levels of oils altered the physicochemical properties and in vitro starch digestibility of extruded pineapple stem starch. The extrudates exhibited poorer expansion and texture characteristics with increasing oil addition, which could be due to the lubricating effect of oils, especially palm oil. The phenolic compounds remaining in all extrudates could inhibit the formation of amylose-lipid complexes (type V resistant starch), as evidenced using XRD, DSC, and FTIR results. However, 10% palm oil or coconut oil can reduce the rapidly digestible starch and increase the resistant starch of extruded pineapple stem starch, which could be beneficial for human health, while a 5% oil addition had no effect on starch digestibility compared to the control extrudate. Further studies on other lipids (e.g., fatty acids) and starch combinations, as well as on the variation of process parameters during extrusion, should be carried out to deepen the findings of this work. Even though the formation of the V-type amylose-lipid complex was not successful in this study, pineapple stem starch can be modified by other treatments to improve its properties and modulate starch digestibility, which may expand the use of pineapple stem starch (an alternative starch) derived from agricultural waste in a wide range of foods in the future. However, the availability of raw materials and the production yield of pineapple stem starch would have to be taken into account for large-scale food production on an industrial scale.

## Figures and Tables

**Table 1:** Physical characteristics, moisture content, and water activity of extruded pineapple stem starch without (control) and with added oil at different levels.

Sample	Expansion Ratio (-)	Bulk Density (g/cm^3^)	Hardness (N)	WAI (-)	WSI (-)	Moisture Content (%)	Water Activity (-)
Control	4.41 ± 0.13 ^a^	0.11 ± 0.01 ^d^	84.04 ± 8.67 ^e^	0.50 ± 0.08 ^c^	0.91 ± 0.01 ^a^	6.77 ± 0.06 ^a^	0.48 ± 0.01 ^a^
PSS + 5% PO	3.14 ± 0.21 ^c^	0.16 ± 0.01 ^c^	134.50 ± 14.46 ^c^	0.60 ± 0.04 ^b^	0.85 ± 0.02 ^b^	6.47 ± 0.76 ^a^	0.45 ± 0.01 ^c^
PSS + 10% PO	2.79 ± 0.09 ^e^	0.27 ± 0.02 ^a^	192.47 ± 30.55 ^a^	0.84 ± 0.01 ^a^	0.73 ± 0.01 ^c^	7.62 ± 0.57 ^a^	0.47 ± 0.01 ^ab^
PSS + 5% CO	3.84 ± 0.09 ^b^	0.13 ± 0.01 ^d^	105.07 ± 16.60 ^d^	0.60 ± 0.03 ^b^	0.85 ± 0.02 ^b^	6.70 ± 0.82 ^a^	0.42 ± 0.01 ^d^
PSS + 10% CO	2.92 ± 0.10 ^d^	0.25 ± 0.01 ^b^	155.64 ± 16.36 ^b^	0.86 ± 0.04 ^a^	0.71 ± 0.01 ^c^	7.29 ± 0.37 ^a^	0.46 ± 0.00 ^b^

Mean ± standard deviation values in the same column that have different superscripts (a–e) are significantly different (*p* ≤ 0.05). PSS, pineapple stem starch; PO, palm oil; CO, coconut oil; WAI, water absorption index; WSI, water solubility index.

**Table 2:** Pearson correlation coefficients between the physical properties of extruded pineapple stem starch without (control) and with added oil.

	Expansion Ratio (-)	Bulk Density (g/cm^3^)	Hardness (N)	WAI (-)	WSI (-)
Expansion ratio (-)	1				
Bulk density (g/cm^3^)	−0.856 **	1			
Hardness (N)	−0.880 **	0.922 **	1		
WAI (-)	−0.782 **	0.923 **	0.901 **	1	
WSI (-)	0.790 **	−0.927 **	−0.908 **	−0.980 **	1

** Correlation is significant at *p* ≤ 0.01. WAI, water absorption index; WSI, water solubility index.

**Table 3:** Total phenolic content and extruder parameters of native pineapple stem starch and extruded pineapple stem starch without (control) and with added oil at different levels.

Sample	TPC (mg GAE/100 g)	Screw Speed (rpm)	Torque (N·m)	Die Pressure (bar)
Native	53.93 ± 0.78 ^a^	-	-	-
Control	50.02 ± 0.79 ^b^	450	40.05	22
PSS + 5% PO	35.69 ± 0.45 ^d^	450	47.90	56
PSS + 10% PO	41.43 ± 1.11 ^c^	450	42.40	28
SS + 5% CO	32.44 ± 0.55 ^e^	450	43.19	60
PSS + 10% CO	39.86 ± 1.10 ^c^	450	41.62	36

Mean ± standard deviation values in the same column that have different superscripts (a–e) are significantly different (*p* ≤ 0.05). PSS, pineapple stem starch; PO, palm oil; CO, coconut oil; TPC, total phenolic content.

**Table 4:** Starch fractions based on in vitro starch digestibility of extruded pineapple stem starch without (control) and with added oil at different levels.

Sample	RDS (%)	SDS (%)	RS (%)
Control	82.97 ± 0.23 ^a^	4.10 ± 1.76 ^a^	12.93 ± 1.85 ^b^
PSS + 5% PO	82.28 ± 1.13 ^a^	3.79 ± 1.94 ^a^	13.93 ± 1.32 ^ab^
PSS + 10% PO	81.24 ± 1.49 ^ab^	3.67 ± 1.51 ^a^	15.09 ± 0.77 ^a^
PSS + 5% CO	82.79 ± 0.80 ^a^	3.74 ± 1.47 ^a^	13.47 ± 1.63 ^ab^
PSS + 10% CO	79.79 ± 1.73 ^b^	4.89 ± 1.50 ^a^	15.32 ± 0.56 ^a^

Mean ± standard deviation values in the same column that have different superscripts (a–b) are significantly different (*p* ≤ 0.05). PSS, pineapple stem starch; PO, palm oil; CO, coconut oil; RDS, rapidly digestible starch; SDS, slowly digestible starch; RS, resistant starch.

## Data Availability

The data presented in this study are available on request from the corresponding author.
